# COVID-19 and the current state of palliative care in the United States

**DOI:** 10.34172/hpp.2022.34

**Published:** 2022-12-10

**Authors:** Ayobami Jadesola Sina-Odunsi, Ayomide Busayo Sina-Odunsi

**Affiliations:** ^1^All Saints University College of Medicine, Saint Vincent and The Grenadines; ^2^School of Medicine, University of Aberdeen, Aberdeen, United Kingdom; ^3^AB Global Health Initiative, Nigeria

**Keywords:** Palliative care, Pandemics, United States, Public health, COVID-19

## Abstract

Palliative care is becoming increasingly pertinent to be strengthened across health systems around the world, and the United States is not an exception. The emergence of the COVID-19 pandemic has disrupted provision and access to palliative care among patients with serious and complex illnesses, critically ill persons, and their families in the United States. Prior to the emergence of the pandemic, the United States faced a number of challenges ranging from racial discrimination, a stressed medical workforce, a lack of passable reimbursement for palliative care, and legal barriers, among others. Unfortunately, these issues have gotten worse amid the pandemic. This further revealed the need to invest more in innovative strategies that will ensure the provision of palliative care services during public health emergencies. In this article, we comment on the current state of palliative care in the United States.

## Introduction

 Over time, technological and demographic alterations such as the aging population, increased life expectancies, varying illness trajectories, and advances in surgical and pharmacological methods have challenged the historical view of palliative (hospice) care.^1^ Palliative care is a dynamic concept that has changed with time in the United States. It has evolved from being a form of care that focused on a person with serious and complex illnesses to encompassing a broader interdisciplinary specialty that looks into both the needs of the critically ill person and their family.^1,2^

 Palliative care is typically delivered simultaneously with all other applicable treatments, including those directed at curing and prolonging life.^2^ At the peak of the pandemic, consultations for palliative care increased four-to sevenfold in some United States healthcare settings.^2^ This study seeks to comprehend the present state of palliative care services in the United States amid the unprecedented coronavirus disease 2019 (COVID-19) pandemic.

## Impact of COVID-19 on palliative care services in the United States

 The arrival of the COVID-19 outbreak introduced weighty challenges to medical providers and healthcare services globally, and the United States was not left out.^3^ Palliative care to patients in need is one of the affected critical services in the United States and its sustainability was threatened by the pandemic among other health services.^4^ The pandemic put an enormous strain on the healthcare system, and almost all medical resources and the workforce were diverted into curbing the spread of COVID-19 in the United States.^3,4^ Preventive measures usher in lockdown and physical/social distancing, some of which affect the supply chain of pharmaceutical drugs and other medical resources.^5^ Families could no longer attend to the care of their sick members due to fear of infections.^4,5^This is of great concern to patients who need palliative care in the United States.^5^

 Furthermore, one of the most noticeable barriers to hospice care during the pandemics was a lack of resources.^5^ Patients were essentially prevented from seeing their healthcare professionals due to staff absences or sick leave, supply shortages, and facilities whose capacity was saturated or super-saturated and unable to admit new patients due to a lack of resources such as bed space and drugs.^6,7^ It has been established that other well-resourced countries, including the United States, have denied more patients who require life-sustaining treatments than have received them.^7,8^ There was a popular perspective on this as an inadequacy of the health system, the COVID-19 outbreak brought an unmatched challenge to the healthcare system. Patients who need sustained palliative care in the outpatient department were enormously affected by the COVID-19 pandemic.^8^ There was a deficiency of approachability to care, especially when the native, district and even state-level lockdowns happen all through the pandemic.^7,8^

 This unprecedented COVID-19 pandemic has spurred the essential need for specialty palliative care. Researchers have reported the use of Palliative Care Quality Collaborative, with a peculiar rapid data collection and analysis.^3^ The findings from a study describe that palliative care use concentrated among older and higher risk patients and challenges to the provision of palliative care during this pandemic. Some of the impact of the COVID-19 pandemic on palliative care services identified in the study include strained communication with patients, family visitation challenges, communication barriers between clinicians and families, rapid changes in palliative care medical management, community care options difficult to find, lack of testing in community-based settings, and guardianship and legal challenges.^3^

 Some healthcare facilities got shut down with services being hampered globally, impermanent, and even longstanding entree to palliative services in the ambulatory care segment becoming imaginary.^9^ Lack of staff and personal protective equipment also affected palliative care agencies as they face challenges in executing in-home services.^9^ The non-existence of nascent tactics for the functionality of the outpatient department during the outbreak became a barrier to access.^5^ The usual stream of clinic patient care and establishing emergency tactics to sustain palliative care in some manners were interrupted.^4,5^

## Recent advances in palliative care services

 Vulnerable populations such as patients that need care and services related to palliative were left struggling amidst the COVID-19 pandemic when specialist healthcare facilities were being shut down at the peak of the pandemic.^5^ Nevertheless, the pandemic brought full attention to the potential of telemedicine in the United States.^9^ Telemedicine, which was formerly bank on occasionally, became the principal platform for healthcare delivery during the pandemic.^8,9^ There was a rapid transformation of palliative care outpatient clinics to telehealth centers.^7^ Concerns were raised about telehealth would be a barrier to multidisciplinary care but amidst the COVID-19 period, the use of telemedicine and visit times were structured similar to in-person visits.^9^ The integration of technology in the delivery of service amidst the pandemic was not peculiar to the health sector or palliative care alone.^8^ Several diverse sorts of practices are moving to telehealth amid coronavirus pandemic. This transition was experienced in palliative care and overall patient volume was maintained.^9^ However, it is important to substantiate that, different factors may serve as a barrier to the utilization of the transited service as not all patients or care can be attended to virtually.^9^ Palliative care and services have precarious psychosocial impacts on the patients and their informal caregivers. Mental health is under-discussed globally and patients who need palliative care may come down with mental issues due to their primary sickness.^10^

 A comprehensive series of health and technology interpolations is being utilized to achieve psychological and social support, augment patient-provider communiqué and advance health outcomes using a patient-centered method.^10^ Evidence from a growing body of scientific works of the literature suggests that digital health tools are progressively utilized in delivering both psychological and social support to patients who need palliative care.^9^ For example, the efficiency of an online support system called Comprehensive Health Enhancement Support System (CHESS) was evaluated using a randomized controlled trial in getting rid of symptom distress among two hundred and eighty-five dyads of patients with non-small cell lung cancer for a period of twenty-five months.^11^ This trial shows that the intervention was found to be meaningfully efficient in plummeting physical symptom distress at four months and at six months emphasizing that digital health interventions can be advantageous among patients who need palliative care.^11^ This poses an opportunity for advancement in this area during public health emergencies. Likewise, and with the development brought by virtual reality software, metaverse, electronic health applications, web 3.0, and software are increasingly used in palliative care settings worldwide.^7,9^

 During the pandemic, the government and health organizations has also introduced advanced healthcare planning that made palliative care services be prioritized.^5,10^ Amidst the barrier posed by the pandemic, advanced care planning tends to remove the barriers and creatively provide care for the highest possible number of patients in need of palliative care.^12^ At NewYork-Presbyterian (NYP), an academic health-care setting with ten campuses in New York City, in response to the increased demand for hospice care, palliative care physician and administrative leadership at NYP piloted multiple creative care models to expand access to palliative care outpatient and inpatient services. The care models included virtual outpatient management of existing patients, embedded palliative care staff, education for providers, multidisciplinary family support, hospice units (which allowed for family visitation), and team expansion through training other disciplines (primarily psychiatry) and deploying an ePalliative Care service (staffed by out-of-state volunteers).^2^

## Equity issues in palliative care and services in the United States

 Equity is a significant value to apply when triaging during a pandemic. Palliative care is a value-oriented method for administering holistic care for persons and their relatives managing serious life-limiting illnesses.^12^ In spite of its proven benefits, access and acceptability are not even across society. In the United States, ethnic and racial minorities and people with lesser social and economic status face interpersonal, infrastructural and health system obstacles to optimum health care, palliative care was not an exception.^13^ As shown by some data, disparities according to race and ethnicity are usually associated with people with lower social and economic status.^12^ In an outbreak, as the health care system becomes further strained, inequities will no doubt become further noticeable.^12,14^ People who are disproportionately marginalized by poverty, historical trauma, discrimination, and language barriers are at a more loss when facing tough decisions about resource distribution.^15^ These patients are aware of this and studies have substantiated that they worry about not getting access to life-sustaining treatments and may be referred to as less deserving of needed care.^12^ Palliative care consequently becomes the considerate option to equipoise this inequity.

 The importance of palliative services in the health system has been recognized, yet substantiation shows economic, social, and cultural factors reduce access to care.^10,13,15^ Disparities amongst socially deprived people (majorly black low-income groups) have been broadly witnessed in contrast with white equals, making these groups at greater risk of hypothetically not getting goal-concordant care; even though it should be noted inconsistencies have also been witnessed among predominantly white rural communities.^12,13^

 Forceful interventions at the end of life have been associated with some black patients.^16^ For instance, intensive care unit admittances; nevertheless, this is time and again regarded as having a deficiency of knowledge about palliative care.^16^ Documented violations of human rights intensify a nous of mistrust at almost all levels of care for certain populations.^16^ Symptom management preferences across cultural groups is a further example popularly cited.

 Integrating hospital palliative service programs inside communities across the United States locally must be ensured for patients to benefit from these services.^12^ Beliefs, attitudes and knowledge are regularly mentioned as causes for dissimilarities in acceptance.^16^ Principal structural factors must also be considered because these elements do impact preferences in the services offered. It has often been mentioned by marginalized populations, such as Black Indigenous and People of color (BIPOC), that adverse happenstance with the health care system due to discrimination and systemic racism are justification for not engaging in palliative care.^16^ As reiterated by some researchers, integration of hospice care into all serious illness is indeed a human right.^1^

## Challenges facing access to palliative care in the United States

 The availability of palliative care services to all patients with serious chronic illness is still highly debated, even in high-income countries such as the United States.^15^ About one-third of US hospitals with over fifty beds lack services that pertain to palliative care.^5^ A current work defined patterns of care in the sole major US health care system in 2012.^14^ It was proven that on average thirty-eight days before death patients received palliative consultation and twenty days before death received hospice care only.^14^

 In order to analyze barriers to palliative care integration, the World Health Organization’s (WHO’s) Public Health Strategy for Palliative Care stands as a framework. Identified barriers to palliative care incorporation across three WHO domains include; Education domain: the dearth of adequate training/education and acuity of palliative care as end-of-life care; Implementation domain: the scarce size of palliative medicine–trained workforce, the challenge of recognizing patients fitting for palliative care referral, and need for cultural change across settings; Policy domain: disjointed healthcare system, need for bigger funding for research, lack of passable repayment for palliative care, and regulatory barriers.^12,15^

 Substantial barriers have been documented as a factor that limits access to hospice and palliative care in rural communities within the United States.^17^ They comprise matters related to educational deficits, differences in cultural values, limitations of the available workforce, geography and supply, and health care system eligibility criteria.^17^ There is a projected palliative medicine human resources for health shortage, this will likely limit growth and prevent many acute care hospitals from actualizing the National Quality Forum’s best practice recommendation that a palliative care package should provide access to palliative care seven days a week and twenty-four hours a day.^18^ Over four thousand physicians presently practice palliative medicine, and the projected current physician shortage is between six thousand and eighteen thousand physicians reliant on the fraction of time practicing palliative medicine.^18^

 Advances in the aptness of referral are not always going in the direction which would maximize the benefits in places where incorporated programs have been established.^18^ For example, British Columbia’s Fraser Health Palliative Care Program caters for a populace suffering about ten thousand deaths a year, including about five thousand referrals each year in diversified sets.^18^ The middling span of stay of patients on the program has fallen from one hundred and eight days in 2007 to sixty-eight days in 2016, with an average span of visit of just twenty-two days.^18^ Researchers from the United States, United Kingdom, New Zealand, and Australia, and certain available data, suggest that this regressive inclination is becoming prominent unswervingly around the globe due to the increasingly more strained palliative care services.^4,17-19^

## Recommendation and Conclusion

 Palliative care services within the United States have experienced swift evolution and transition within the past decade.^12^ There is no doubt that COVID-19 had an impact on various segments of the health care system, including palliative care. Among other things, there is a constant need to improve competence in order to provide patients with multiple options for receiving palliative care at home, particularly during pandemics and outbreaks. Prospective telemedicine practice/application trends during pandemics and beyond should be investigated further. Future palliative care services should also emphasize the use of cutting-edge care practices that support social distancing while improving the consistency of care, including the expanded use of telemedicine. Factors such as poor internet access and technicalities involve in using health-based technologies should be considered during implementation. This reifies need to integrate palliative care into pandemic planning.^5^

 During outbreaks, equity is an especially important value to emphasize in service delivery.^20-22^ It is important to note that even in the best of times, some vulnerable populations who are fundamentally vulnerable or who suffer from severe illness face significant difficulties and inequity in accessing health care. Patients who require palliative care while also experiencing disparities in social factors of health face additional challenges in obtaining care. To ensure health for all, services during and after pandemics should be based on equity, with all potential biases eliminated.

## Acknowledgements

 We thank Yusuff Adebayo Adebisi for the editorial assistance.

## Author Contributions

**Conceptualization:** Ayobami Jadesola Sina-Odunsi, Ayomide Busayo Sina-Odunsi.

**Writing – original draft: **Ayobami Jadesola Sina-Odunsi, Ayomide Busayo Sina-Odunsi.

**Writing – review & editing:** Ayobami Jadesola Sina-Odunsi, Ayomide Busayo Sina-Odunsi.

## Funding

 None.

## Ethical Approval

 Not required.

## Competing Interests

 We declared no competing interest.
